# Physical Education

**Published:** 1833

**Authors:** 


### Art. V.—Physical Education.

*Remarks on the Influence of Mental Cultivation upon Health.* By Amariah Brigham. Hartford, C. 1832.

*First Annual Report of the Society for promoting Manual Labor in Literary Institutions, including the Report of their General Agent,* Theodore D. Weld. New-York, 1833.

Physicians have two great classes of professional duties—to prevent diseases, and to cure them. To these might advantageously be added a third—to collect their debts; but we shall not insist on that at present—nor even discuss the curative department. Our object is the preventive, or to give it a higher dignity than can be conferred by a Latin cognomon—the *Prophylactic.*

It has, however, been seriously doubted whether prophylactic medicine, can be cultivated without abridging the profits of the profession. The venerable and thrifty adage—"an ounce of prevention is better than a pound of cure," cannot, say these sceptics, by any kind of logic, be shown to connect itself with both patient and physician. To the former, continue they, the word 'better' exclusively applies, and, therefore, 'twere better for the latter to eschew the maxim. This argument deserves grave consideration; but having, perhaps hastily, "made up our mind," that the interests of the faculty are identified with those of society, we shall proceed in the affirmative; still retaining the negative, however, among the *jura reservanda* of the profession, to be called up if occasion should require.

Assuming, then, for the present, that the faculty should labor to preserve society from disease, we proceed to solicit the attention of both, to the subject which is suggested by the works prefixed to this article.

Not limiting ourselves to the influence of labor as a means of preventing disease in students, and young persons generally, we propose briefly to consider the philosophy of physical education at large.

Intellectual education, is the exercise of our inquiring and reflecting faculties—moral education, of our sentiments, affecttions, and propensities—physical education, of our bodily organs. To attain the condition so beautifully expressed by the ancients, *mens sana in corpore sano*, the whole of these varieties of exercise or education, must be carried on at the same time. If any organ or faculty, either of body or mind, be neglected, it falls into debility and disorder and never fails to react on the others and limit their perfectibility.

In simple and rural states of society, education is chiefly physical. The body is well developed, hardy, and comparatively free from disease; but the mind remains uncultivated and inefficient. In civic and highly civilized society, on the other hand, the body is neglected, and becomes a prey to the active and over excited faculties of the soul. Thus *inertia* of body and overaction of mind, are two great sources of disease in civilized communities. Still, notwithstanding men's maladies both of body and mind are more numerous in the literary; than in the hunting, pastoral, and agricultural states, the race multiplies in a greater ratio; and the examples of a protracted and cheerful old age, are proportionally more numerous. Were this not the case, civilization would be a curse instead of a blessing.

The problem involved in this statement, is not difficult of solution. Civilization multiplies diseases, but it, also, multiplies remedies, both preventive and curative; and according to the ratio in which these are developed, will be the happiness of society, so far, at least, as relates to disease, both of body and mind. Here then we find one of the great elements of the usefulness and dignity of the medical profession, and are enabled to perceive, that but for its helping hand—the *tactus eruditus*—society could not continue to advance in civilization. But let us come up to the business which lies before us.

Physiology will in vain attempt to lift the veil, which conceals the union of mind and body. It cannot reveal to us how the moral is combined with the physical; how ideas and motives are connected with atoms, having magnitude, density, weight and other mechanical properties. But we *can* observe the effects of the varying states of the sentiments and faculties of the mind, on the matter of which our bodies are composed; and, inversely, perceive the influences which the different corporeal conditions, exercise over the mental faculties. Thus we can ascertain the laws of their reciprocal action, and these constitute the philosophy of physical education.

The body is not only the machine by which the mind acts, in most of the duties and pursuits of human life, but its domicil—its home; and in proportion as this is well ordered and comfortble, *cæteris parabus*, will be the serenity, activity, and efficiency of the mind.

The body, however, is not merely the habitation of the mind and the machine by which it acts on other bodies, but the medium through which it acquires a knowledge of their properties and movements. The senses are the inlets of all our knowledge, and the impressions made on them are purely physical—that is, the action of matter upon matter. To have correct information from without, the organs of sense must be well developed, properly disciplined, and free from disease. The impressions must be transmitted to the mind through the nerves and brain, but to do this successfully, they must be kept in a state of health.

When children are reared in the country and study but little, they generally subsist on a simple diet, keep regular hours of sleep, and take abundance of exercise, in the open air. Thus trained, their bodies are well developed, and morbid sensibilities—the pains and penalties of violated physical laws—are almost unknown.

The causes which, in civilized society, interrupt the proper development, and disorder of the functions of the body, in children, young persons, and students, may be divided into *physical* and *moral*. Let us, *seriatim*, review the most prominent.

1. *Absurdities and excesses in diet.* The genius of civilization has not been backward in divising the means of a diversified gratification of the palate. He has even invented a name for the science of eating; and the gastronomer aspires to take rank with the philospher. A thousand modes of regaling the appetite have been invented. The earth, the waters, and the air, have been ransacked for the raw materials, and all the arts of the kitchen laboratory, all the science of the professional cook, have been exerted to manufacture them into compounds, which are sweet to the taste, but to the belly, bitter. When children and students, taking but little exercise, are pampered on these compounds, their sensibilities are rendered morbid, their digestion deranged, and their bloodvessels over-distended. Irritations are set up in the stomach, lungs or brain; and the individual, literally eats himself into the hands of the doctor and the druggist; from which he seldom escapes without new desolations of purse and constitution.

2. *Abuse of stimulating drinks.* Children and young persons should drink milk or water. Their excitable systems are irritated by whiskey, brandy, wine, ale, and even cider undiluted. The same remark, is applicable to most students, before the age of forty. Children are injured by tea or coffee, much more than by alcoholic drinks, because they are much more used. The mother, who drinks those infusions, with comparative impunity, seldom comprehends why they should injure her daughters. It is for the family physician to disabuse her mind of this error, and thus preserve her fair offspring from the disasters of excessive stimulation. Students commit upon their physical systems, the most deplorable outrages, with these facinating stimulants. No other drinks impart such excitement to the feelings and faculties of the mind. To the sprightly they are nectar; to the dull the waters of Pierus;—who under their influence, rise like Dædalus, and soar for an hour on the wings of genius. But all this excitement of the soul is purchased at the expense of the body, especially in early life, and in those of a delicate and excitable temperament. The male sex may take them with more impunity than the female; the sluggish with less injury than the sensitive; and the aged with an advantage they never confer on the young. When the latter indulge in them, they violate a law of their physical systems, and the punishment is a black train of nervous disorders. The short and sunny day, is followed by a long dark night of pain and despondency.

3. *Abuse of Tobacco.* Tobacco is a narcotic poison, against which the *vis conservatrix naturæ* exerts her utmost power. When she yields, and the appetite is modified to the offensive qualities of the deleterious plant, the desire for it becomes importunate and uncontrollable. The contest between nature and fashion takes place in early life, and then it is, that the system—endowed with acute and delicate sensibilities—is most injured, by the impress of the poison. It is a great mistake, that the spitting, for which we are so renowned, and which is promoted by tobacco, is the chief cause of bodily injury from its use. It acts upon the whole nervous system, producing tremors of the muscles, and weakness and acidity of the stomach. To the phlegmatic it is least prejudical, and many persons, after the age of forty, derive comfort from the pipe and quid. But their sinister effects in early, far outweigh all their aggregated benefits in after life, and call loudly for a total proscription. In this case, as in that of most other vices, reformation must be dictated by the old, but commence with the young. The former will not give up the habit, but the latter might be preserved from it. Public sentiment should frown upon it; fashion look with contempt, and sarcasm utter its sneers. Masters, parents, and all who have the care of boys, should forbid the use of the noxious plant: and it ought to be a fundamental law of every academy and college, that no young man should be retained within its walls, who indulged in the loathsome and pernicious practice; lastly the physician should interpose his authority, and point out to the youthful imitator, that victory, in the struggle to which fashion invites him, would consist in his total defeat—in the triumph of nature, over the invasion of an absurd custom.

4. *Absurdities in dress.* These begin with our existence. The young infant is compressed in swaddling clothes, which often bind the trunk of its body like a well-hooped keg. Thus a proper play and development of the viscera is prevented, and a foundation laid for disorder in their functions. The brain on which the heart is incessantly pouring such torrents of blood as predispose it to disease, is confined in its own heat with caps by day and by night, throughout summer as well as winter. Before the child is able to take the floor, its feet are encased in shoes and stockings; and when it can crawl abroad, they are never suffered to touch the earth, whose impress is almost as necessary to the healthful and perfect growth of man, as the worm on which he tramples. All children delight to go barefooted on the ground. It is one of their instincts—they flourish well under it. Indulged in this exposure, their feet are better knit together and stronger, they are more likely to escape that fundamental scourge of civil society *corns*, they run faster, jump higher, and take more exercise, they are less liable to colds and croup, and as they grow up are less obnoxious to consumption. Mr. Locke advised that children should wear shoes with holes in them. Let the rich observe the advice of this great philosopher wh studied man from soul to sole.

Did our daughters escape from the trammels of custom when they escaped from their "leading strings," one source of our professional income would be dried up. But fortunately for the faculty, a time arrives, when fascinated by fashion, as the sparrow by the serpent, they yield themselves up and walk deliberately into new shackles, prepared, and brandished to their view, by maternal affection. Now comes the bed of Procrustes—not however, to bring all our daughters to the same length, but the same breadth. The shaft of the animated column must be compressed in the middle; its proportions improved, till it shall approach the *beau ideal* of the *beau monde*, and captivate the *beaux*. What does it signify, if the stomach and lungs and heart like plants, sprouting beneath logs and stones, should germinate in new and unnatural directions, or be arrested in their growth, or fall into disease—will not the end justify the means? Who would wish to grow up according to nature, when art has wrested the sceptre from her hand? Who so grovelling as to prefer sound health to the admiration of the fashionable world? Who would not violate the laws of nature rather than those of civilized society? Who would not prefer sickness and premature death, to the criticisms of the *haut ton?* But to be grave, compression of the chest from twelve to eighteen, is a prolific cause of dyspepsia, palpitations of the heart and consumption; of which all parents should be warned, and against which every physician should raise his voice. The great organs are not only more or less displaced, and impeded in their functions, but from the difficulty of making deep inspirations, a due degree of exercise cannot be taken and the individual suffers all the superadded effects of idleness and inactivity.

5. *Irregularities in sleep.* Children require more sleep than adults. The ambitious and excitable should not be permitted to abridge the necessary hours of sleep, for the prosecution of their class studies. They should never sit up to a late hour for this purpose. They study much better and with less injury to their constitutions, in the morning than at night. To retire early and rise early, is the law and inclination of childhood, which the usages of society frequently contravene. Young men devoted to study, frequently perpetrate the same violation, and never escape the penalty—weak, watery and inflamed eyes—head-aches—indigestion or irritability. The emulous youth should be admonished, that he cannot scale the heights of Parnassus by midnight assaults. Even the Ogre-boots of fairy land, would avail him nothing, if drawn on during the hours which nature has consecrated to rest. Repose favours renovation—and sound sleep exalts vigilance. Taking life throughout, as many hours should be spent in sleep as in study. In childhood and youth more, in old age more, in manhood less—but the student should never persevere when nature flags and yawns with exhaustion. The richest heritage of man, is a sound constitution: one of his greatest follies, its early desolation.

6. *Immersion in a confined Atmosphere.* Close confinement is abhorrent to the taste and constitutions of children and youth.

It is scarcely less pernicious in middle life. Even in old age it is prejudicial. Fresh and pure air is one of the essential supports of human life. It acts as a general stimulant on the surface of the body, and of the lungs and ærial passages; it defecates the blood, and through the medium of that fluid animates every part of the living mechanism. All students and literary men should expose themselves daily to the breezes of the fields and woods, where, in common with the plants among which they stray, they will inhale the "breath of life," and return to study with freshened energies of body, and brightened faculties of mind. Dr. Franklin has left us a paper on the *fidgets*, for which he advises exposure of the body to a cool and fresh air. The student who follows his example, may hope to equal him in health if not genius, learning and fame.

7. *Neglect of Exercise.* Motion is the great law of nature. Rest is found no where on the surface of the earth. The air and waters are in perpetual agitation, and even the soil is a great laboratory, in which atoms of matter are unceasingly acting on each other. The living plant has its intestine movements, and takes exercise in the winds. The animal kingdom presents two kinds of action—internal and locomotive; the former occurring in the apparatus of nutrition, the latter in the organs of external relation. They reciprocally sustain and quicken each other. The suspension of the latter constitutes apparent, of the former, actual death. When their momentum is greatest—provided they are regular—the individual enjoys the soundest health, feels happiest, and is most efficient. When the internal movements are weak he is debilitated—when irregular diseased—in both cases the external, are reduced in activity and power. On the other hand, when these are defective, the internal speedily languish, and this languor reacts on the locomotive. Hence we deduce the physical necessity of exercise.

Parts which are not exercised, like faculties of the mind not educated, grow imperfectly—perform their functions awkwardly—and readily fall into disease. If the slothfulness be universal, every part is feeble and inefficient. Imperfection is stamped upon the whole machine. The dandy of the drawing room, cannot expand into an Apollo Belvidere, nor the sluggard into a Hercules. Animated grace and living strength, can come only from exercise. Thus all who aspire to beauty symmetry and power of body, should take diversified and laborious exercise. The muscles which are most exercised grow fastest—have most firmness—and the greatest facility of movement. Ease and adroitness come only from appropriate exertion. The senses are under the same law. If not trained by occupation, they are defective in sensibility, and the movements necessary to their efficient action. But let us look at the influence of locomotive movements on those of the viscera. This influence is chiefly exerted through the great function of respiration. In exercise the diaphragm and abdominal muscles compress and agitate the organs of digestion—whereby the portal circulation is accelerated, the bile ducts emulged, and the peristaltic action of the stomach and bowels promoted. Thus digestion, chylification and elimination, receive a salutary impulse. Those who take active exercise will have little need of Mr. Halstead to knead their abdomens. Exercise quickens the venous circulation, and returns the blood more rapidly on the heart. The great "forcing pump" thus stimulated, waxes strong and gives to the circulation the momentum necessary to "deeds of daring." The blood, being impelled more copiously through the lungs, those organs expand, and the chest enlarges; the pulmonary vessels acquire a tougher texture and become more firmly knit together; thus hæmorrhages are warded off, and consumptions, asthmas and dyspnœas set at defiance. Meanwhile the blood is defecated of its impurities, which circulating throughout the system as a poison to all the organs—irritate their sensibility and depress their powers. Hence, in a great degree, the cheering and salutary influence of a single hour of active exercise in the open air, when the sedentary student feels restless, nervous and fidgetty. In a similar way exercise, drawing and impelling the blood into the skin, maintains the function of perspiration, and still further purifies the vital fluid; while it promotes the generation of animal heat, and renders foot-stoves and fall-baths, the common resort for cold feet, entirely superfluous. In addition to all this, exercise increases interstitial absorption, and removes the decayed elements of the living fabric to make room for new ones;—thus giving sweetness to the flesh, and limiting the generation of fat. Of all the nostrums for corpulence, exercise is, indeed, the only one which needs not the aid of certificates and affidavits.

Finally, exercise exerts upon the brain and the nerves of sense and motion the most salutary influence. It prevents stagnations and congestions of blood within the skull and spinal column, and supplies the nervous matter with that which has just been purified in the lungs. It conducts the "vital fire" from the brain into the muscles—diffuses it according to the great laws of the system--preserves the viscera from conflagration, and the brain itself from the withering consequences of its concentration within the delicate mechanism of that great organ. Thus exercise prevents or cures headaches and vertigoes--breaks up and disperses many of the clouds which, at times, hang over the affections, and detaches the films from the "mind's eye"—rendering the confused palpable and the obscure bright.

It is difficult to lay down particular rules for exercise. The following deserve attention, but every physician should prepare himself to give correct advice, on this as on every other part of hygiene.

1. The younger the child the more time should be devoted to play and the less to study.

2. Play and every kind of exercise should, as far as possible, be carried on in the open air, and not limited to fair and dry weather.

3. The child should be supplied with some immediate motive; its mind cannot embrace the remote one—the improvement of its health and constitution. Even grown up children, as all experience shows, must have some other incentive than the dread of disease and doctors' bills to induce them to take sufficient exercise.

4. Exercise should be so diversified as successively to bring every muscle into action.

5. It should be taken daily, and carried so far as to induce perspiration and fatigue.

6. It should not be taken immediately after meals—especially dinner.

7. The more precocious the intellect and the more delicate the bodily frame of the child or young person, the greater is the necessity for exercise;—the more pernicious are confinement and protracted study.

8. Parents and teachers should carefully distinguish between mere relaxation from books and active exertion. Hard study for four hours a day, without exercise, will do more harm, than eight hours with it.

9. It is equally necessary to both sexes.

10. It should be continued to old age, when it is often highly beneficial, but should then be of the passive kind. The aged are not locomotive, and should be exercised.

To enumerate all the imperfections and infirmities of body, which spring from absurd modes of life and neglect of exercise, by the young and the studious, would make a "black book" as large as Mrs. Royall's, which, however, we have not seen; and could not fail to scandalize many of our friends of either sex, we shall therefore drop the matter short, and proceed to the influence of moral causes on the constitutions and health of the young and studious.

With every progressive step in civilization, the mind acquires greater influence over the body. The stirring, sturdy and illiterate young farmer of Ohio, the "hoosher" of Indiana, and the "leather-stocking" of the entreme frontiers of Illinois, Missouri and Michigan, grow up, flourish and decay under the influence of physical causes, like the tall grass of the prairies or the hardy oaks of the forest, among which they pursue their simple occupations. With minds as little cultivated as the fields of the hunter, and but few of the passions called into play, their iron nerves are seldom made to vibrate, by the intense intellectual excitement, and the 'thousand and one' deep and swelling emotions, which agitate and enfeeble the sensitive nervous systems of the scholar and the child of luxury and indulgence. To register the various ills which "flesh is heir to" from excessive cultivation of the faculties and emotions of the mind, without adequate bodily exercise, would exceed the limits of the longest and dullest review. We shall decline the ungrateful task; and, as physiologists, proceed to inquire into *modus operandi* of intellectual culture, on the physical systems of those who are too studious, or, what is far more common, too indolent to take exercise.

Cultivation of the mind, is attended with exercise of the brain. When carried too far, without exescise of the body at large, the whole nervous system is rendered morbidly sensitive, and the influence which animates it, swells and revels in the mechanism, like electricity among the summer clouds at noon day, which send forth awful explosions, with but few visible flashes. In this condition the patient, for such he really is, finds every variety of painful sensation compressed into a single hour; or feelings of gloom and despondency, chace each other like the waves of the dead sea.

In this state, almost the whole excitement of the body is accumulated in the nervous system; which notwithstanding the monopoly, performs its duties imperfectly, because its movements are disordered. Hence the various functions fall into confusion, and every organ is more or less diseased. The liver secretes but little, or a vitiated bile; the bowels become torpid; the stomach fails in its action; and the enfeebled heart struggles and palpitates under its load.

But of all the organs the brain itself suffers most. Like every other part, when irritated an unwonted determination of blood engorges its vessels—the delicate pulpy matter is compressed—too much heat is liberated, and inflammatory actions are set up. Thus maddened, it sends forth the mandates that awaken epilepsy, chorea hysteria, spasms and neuralgia; or withholds the genial influence necessary to firm muscular motion, and debility, paralysis, or apoplexy is the consequence. Should none of these maladies arise, its functions, as the organ of feeling and intellect, may disclose the internal agitation, in the various frightful forms of petulance, fickleness of temper and purpose, irresolution, hypochondraism, melancholy, fatuity, mania and suicide.

Extreme cases of this kind are certainly not common; but no observing physician has failed to note a great variety of nervous affections, which manifestly arose from the moral causes we are now considering.

The friends of education especially in the eastern states, might almost be classed with the *causæ morborum remotæ.* They have enlarged the sphere of school studies, multiplied the variety of books to an indefinite and perplexing extent, allured the inquisitive by an endless succession of novelties, impelled the ambitious by stimulating the spirit of rivalship; and made hard study not merely a personal and social, but a moral duty. They would elevate society with a high pressure engine, and hang heavy weights upon its safety valve. Their object is noble—their motive exalted—their benevolence glowing, but they have too often overlooked the physical consequences of the application they would inspire, and seem not constantly to have remembered, that many of the gifted, but delicate and weakly, must perish prematurely under such intense and diversified study; while all should have bodily, proportionate to their intellectual exercise, or they can neither form strong constitutions nor preserve sound health.

One of the great faults, the greatest perhaps, of modern education, is, that it depends too much on books and too little on observation; and one of the greatest misfortunes of the modern student, is, that the progress of science has given him too much to study. But however great may be increase of books, the student should never lose sight of the momentous truth, that he can only study them with success, while he preserves his health unimpaired; and that to do this, he must invigorate his frame by recreation or labor in the open air. Such was the method of the ancients; and it may not be unprofitable to inquire for a moment, into the causes which have superseded it, with methods so pernicious to health.

The first which occurs to us, was the overthrow of the Roman Empire. Under this catastrophe, learning and science took refuge in the cloister, and was protected by religion. But the anchorite was sedentary and inactive; and when he restored the heritage to modern Europe, it came as something to be enjoyed and cultivated, by patient and protracted sittings before the shelves of the book case, and no where else.

The next cause in order, was the application of gun-powder to military operations; by which science was substituted for bodily strength, and lost its personal character. Thus it was no longer necessary to educate heroes to feats of muscular power, and the physical became still more widely divorced from the intellectual.

In the third place the art of printing, has multiplied books, inspired a taste for reading, withdrawn us from the study of nature by observation abroad, and immured us within the walls of academies and colleges.

Lastly, the lapse of time and the progress of discovery, have enriched the archives of knowledge with vast treasures of mathematical and natural science, which can not be unlocked and examined, without patient and arduous labor in the observatory, the closet and the laboratory; so that of necessity, the modern student leads a more sedentary life, than the ancient.

What other causes may have contributed to dissolve the natural and necessary connexion between study and exercise, or the education of the body and the education of the mind, we need not stop to inquire. The fact of their separation is as indubitable, as its consequences are disastrous to the student of the present day. Let us indulge in a brief review of the advantages and pleasures, enjoyed by the students of antiquity, and contrast them with the confinement, the deprivations and bodily imfirmities of our own age.

The motives for promoting a good development and preserving health and vigor, in ancient times connected themselves with the great functions which men were called upon to perform in society; while the methods by which they of necessity acquired their knowledge were favorable to the same objects. Having, as we have seen, when compared with the moderns, but few books, they supplied the aliment of thought by observation, and the practice of observation kept them active. The great map of external nature lay unfolded before them, all fresh and beautiful from the hand of the Creator—they traversed its untrodden wilds—clambered up its frowning and unscaled precipices—descended into its deep and unexplored valleys—meandered its streams, as they murmured through vast and unpeopled solitudes—marked the external features of general nature—listened to the chorus of the animal kingdom, from the bee of mount Hymettus to the lion of Abyssinia—inhaled the fragrance of the vegetable world, and feasted their vision on its forms and colors, in a region, which, more than any other, combines, the useful plants of the north with the luscious fruits and beautiful flowers of the south—their eyes wandered among the clouds and noted the forms and colors, which portend changes in the constitution of the air, the causes of which philosophy had not yet revealed—beyond the clouds they counted the stars, and gave names to the various constellations—finally, turning upon themselves, they studied man in society as he then was, not as he had been, by observation instead of historical research, made him display himself in *action* through all the stages of life, and looked on, applauded and registered his feats, in the Elian, funeral and Neptunean games, in the gymnasium, the portico and the grove—in the arena, the circus, the forum and the senate! Philosophers then travelled on foot from city to city, as a means of that improvement, which is now sought by crawling from alcove to alcove of our mouldering libraries—inhabiting a genial climate, they read and conversed, and thought, in the wide and moving air—the teacher then assembled his pupils around him in open colonnades, or under the shade of majestic and embowering trees—where they breathed a pure atmosphere—while the fanning breezes kept down that fever of the brain, which consumes the student of modern days!

What a luxury study must then have been, compared with that of the school house, where children are compelled to sit *still* (an outrage on their physical nature,) for six or eight hours of the day on wooden benches, *scrowghing* each other, in a hot and confined atmosphere, and committing to memory the language and literature of the nations, who gathered together from the great field of nature, the heritage which has *unfortunately* descended to posterity. Had only their methods of study reached us, the constitution both of mind and body in modern times, would have been superior to what it now is. We have, let us repeat, too many books—and rely too much on reading—they consume more time than can be spared from exercise and observation—they substitute for our own, the observations and reflections of other minds—they present us with results which should be attained by our own inquiries. The mind does not grow healthfully on food that has been digested by a hundred generations—we should observe the phenomena of the natural and moral world for ourselves—compare them for ourselves—and draw conclusions for ourselves. We should consider book studies as but a part of our education—the rising generation, male and female, should be encouraged and required, to exercise their senses on the qualities of all surrounding bodies—in the waters, on the earth, and in its caverns and recesses—in the atmosphere and in the physical heavens—they should be incited to exercise their muscles, in walking, climbing and riding—in archery—in the play ground—in hunting and fishing—in searching out, collecting and preserving minerals, plants and animals—in gardening and husbandry—in various mechanic arts, and philosophical manipulations.—In all these pursuits, while their limbs would expand in strength and beauty, their organs of sense would be rendered acute, and their minds enriched with new ideas. Thus raciness and originality of character would be secured; for to have these the mind must drink for itself, at the pure and sparkling fountains of nature.

In evidence of this, we find that the *country* and *villages*, have furnished a great proportion of the distinguished original genius of the world. It is true, that the mass of the people reside in these instead of the cities; but it is equally true, that the majority of our seminaries of learning are in or about the latter; and if these were the only sources of intellectual nourishment, the cities should rear up all our great men. Such being far from the fact, we feel strong in the conclusion, that the occupations, the pure air and water, the simple diet, the natural hours of meals, of sleep and of labor, the natural scenery and the natural events of rural life, are equally favorable to the growth of mind and body. Under such circumstances, both are less fettered and compressed by artificial usages, and tyrannical superintendence, than in cities; where a thousand rules and regulations, unneeded and unknown in the country, are absolutely necessary, to enable the dense masses of population to abide each other's movements—to stand up against each other's intellectual elbowings.

The youthful civic mind is more scholastic and disciplined—more amenable to precedents—deeper fraught with learning. When a problem is to be solved or an object accomplished, it seeks for the means amidst its stores of book acquired knowledge, and should it find none, knows perhaps no other resource:—the young mind of the country, draws its intellectual treasures from the earth, the woods and fields—nature is its schoolmaster, all her objects its books—it roams at large over her works, and undertakes the solution of the little problems which she offers at every step. It may solve none of them correctly, but that signifies nothing—it is exercised, it contemplates the relation of cause and effect—it thinks—it thinks by itself and for itself. Or, passing from natural problems to ends and objects for attainment, it must devise for itself the means of their accomplishment, and these it must invent and apply, unaided by precedent or even elementary instruction. Thus from necessity, it becomes self-dependent, resolute, inventive, and original. Its learning may be small, but knowledge is the *desideratum*, and its knowledge is great. It acquires, at least, a taste for executing in its own way, and an ability for inventing and applying new means to the accomplishment of new objects, the strongest sign and highest attribute of useful genius.

Let such a mind, sustained by the frame and energies of a body which has been knit together amid the snows and storms, and piercing winds of a healthy country, be, in due time, subjected to academic and civic culture, not carried so far as to enervate either the mental or corporeal powers, and we shall not be disappointed in the fruits of genius it will bring forth. They will indeed be the products of a sound and cultivated mind in a sound and vigorous body.

Men and women who have been *thus* educated, are no hot house plants, leaning against the wall, and requiring to be watered—blooming but in one degree of heat—prone to disease in the midst of a thousand artificial excitements—shrinking from every blast—wilting after every frost, and dying when exposed to the storm:—but the green trees of the lofty forest—the earth's plumes and pride—requiring no foster mother but nature—raising their voices amidst the tempest—recruiting, beneath the ice of winter for new displays of vigor and beauty in spring—throwing back the burning rays of summer—shedding a copious harvest of fruit in autumn, and perishing but from age.

But let us return from declamation, to the useful facts and sound common sense of the works prefixed to this article.

The object of Dr. Brigham's book, is to disclose the pernicious influence of forced mental culture, precocious ambition, and inflamed curiosity, on the health and constitutions of children, young persons, and students generally. The first section presents the brain as the material organ by which the mental faculties are manifested, and abounds in just views of its physiology. Coinciding with the best writers, Dr. B. regards insanity as the consequence of morbid action in the brain, but this may be and often is, the consequence of moral causes.

"Insanity furnishes further evidence that the brain is the organ by which the mind acts, for this is not a disease of the immaterial mind itself, but of the brain, resulting often from some injury. Such a diseased state of the organ of the mind, of the very instrument of thought or of some part of it, deranges the intellectual faculties, just as a diseased state of the stomach deranges digestion. The immortal and immaterial mind, is, in itself, surely incapable of disease, of decay and derangement, but being allied to a material organ upon which it is entirely dependent for its manifestation upon earth, these manifestations must of course be suspended or disordered when this organ is diseased."

"I might adduce many more cases to prove the very intimate connexion between the brain and the mind, and that it is a defective brain which makes the idiot, a diseased brain which causes delirium and insanity; and that all the various states of mind produced by alcohol or by opium, &c., arise from the disordered action in the brain which these articles produce; that the weak mind manifested by the infant, or the feeble mind by the aged, is produced by a small, undeveloped, or enfeebled and diseased brain, and not by a change of the immaterial mind itself. But cases enough have been cited to prove these truths. And if we do admit them, and consequently that the brain is the organ by which the mind acts, all will acknowledge the necessity of guarding this organ most carefully, of exercising it with extreme caution, of not endangering its delicate structure at any period of life by too much labor, or prevent its full development by too little; for the regular exercise of all the organs of the brain is necessary to prepare them for the active and powerful manifestation of the mental faculties.

"The healthy condition and proper exercise of the brain, is therefore far more important than that of any other organ of the body, for we might as well expect good digestion with a diseased stomach, or good music from a broken instrument, as a good mind with a disordered, enfeebled, or improperly developed brain. And yet, how little regard has been paid to these important truths, in the cultivation of the mind! While people are exceedingly fearful of enfeebling and destroying digestion, by exciting and overtasking the stomach, they do not appear to think they may enfeeble or derange the operations of the mind by exciting the brain, by tasking it when it is in a tender and imperfectly developed state, as it is in childhood."

In his second section, our author discusses the condition of the brain in early life, and the effects of its excitement and unnatural growth on the manifestations of the mind. In infancy and childhood the organ is soft, exceedingly vascular, and grows rapidly. Hence it is tender, excitable and prone to disease, beyond the other organs of the body.

"It is, therefore, deeply important, that the natural action of the nervous system should not be much increased, either by too much exercise of the mind, or by too strong excitement of the feelings, lest at the same time the liability of children to nervous diseases be increased, and such a predominance given to this system as to make it always easily excited, and disposed to sympathize with disorder in any part of the body; giving a predisposition to hypocondriasis and numerous and afflicting nervous affections.

"Mental excitement as has been shown, determines an unnatural flow of blood to the head, and increases the size and power of the brain, just as exercise of the limbs enlarges and strengthens the muscles of the limbs exercised. The wonderful powers of mind which an infant or child sometimes manifests, and by which he surpasses ordinary children, do not arise from better capacity in the mind itself of the child, but in fact from a greater enlargement than usual, of some portion or the whole of the brain, by which the mind is sooner enabled to manifest its powers. This enlargement takes place whether the mental precocity arises from too early and frequent exercise of the mind, or from disease, and it must arise in one of these two ways. But in my opinion, mental precocity is generally a symptom of disease, and hence it results that those who exhibit it die young. This fact ought to be specially remembered by parents, some of whom regard precocity, unless accompanied by some *visible* disorder, as a most gratifying indication; and on account of it task the more the memory and the intellect of the child. Sometimes, however, it is accompanied by visible alterations of the head, and then the fears of parents are greatly awakened. Take for instance the disease known by the name of rickets. Every person understands that this is a disease of childhood, and according to the best medical authorities, it arises from the irritation or inflammation of some organ, and frequently of the brain. Its most characteristic symptoms when it affects the brain, are an enlargement of the head, and premature development of the intellectual faculties. On examining the heads of those who have died of this disease, the brain is found very voluminous, but ordinarily healthy."

Every physician, as our author has done, must have observed in children affected with disease in the hip joint or spine, which is generally or always, attended with a *scrophulous* diathesis,—a similar precocity of the intellectual faculties and moral sentiments. When such children die, their loss is deeply deplored, *because* they gave such great signs of bright genius and pure morality; but these early developments are anticipations of the future, and no more foretoken great intellectual power, in coming manhood, than a hypertrophy of the heart is auspicious of a vigorous circulation; or the rapid growth of a young tobacco plant from the ashes of a Kentucky brush-heap, presages a stalk thirty feet high. The same remark is almost equally applicable, to the hypertrophy of the infantile mind, from excessive education. We every day see adults deformed by spinal disease in infancy, who manifest no mental superiority; and quite as often, find the brilliant intellect of childhood, obscured at full maturity, by minds which had been pronounced dull and common place.

The following remarks which close the section we are passing over, are eminently deserving the attention both of parents and preceptors. They contain much sound physiology.

"The activity of most of the organs of the body can be very greatly increased; they can be made to perform their functi ns for a while with unusual facility and power. I will dwell upon this fact a little. The child, for instance, may be gradually accustomed to eat and digest large quantities of stimulating animal food. I have seen an instance of this kind, and when I remonstrated with the parents on the impropriety and danger of allowing a child but two years old, such diet constantly, I was told that he was uncommonly robust; and indeed he appeared to be in vigorous health; but soon after this he had a long inflammatory fever of an unusual character for children, which I attributed at the time, to the stimulating diet allowed him. This diet appeared also to have an effect upon his disposition, and confirmed the observation of Hufeland, that "infants who are accustomed to eat much animal food become robust, but at the same time passionate, violent and brutal." A child may also be made to execute surprising muscular movements, such as walking on a rope, and other feats; but these are learned only by long practice, which greatly developes the muscles by which the movements are executed. It is from this practice, from frequent and powerful action, that the muscles of the arms of blacksmiths and boxers and boatmen, and those of the lower limbs of dancers, and those of the face of buffoons,* become strikingly enlarged when compared with the muscles in other parts of the body. Every employment in which men engage brings into relatively greater action particular parts of the system; some organs are constantly and actively exercised, while others are condemned to inactivity. To make therefore one organ superior to another in power, it is necessary not only to exercise it frequently, but to render other organs inactive, so as not to draw away from it that vital energy which it requires in order to be made perfect.

"The important truth resulting from these facts, that *the more any part of the human system is exercised, the more it is enlarged, and its powers increased*, applied equally to all organs of the body; it applies to the brain as well as the muscles. The heads of great thinkers, as has been stated, are wonderfully large; and it has been ascertained by experiment, that they frequently continue to increase until the subjects are fifty years of age, and long after the other portions of the

*People who are in the habit of indulging anger, very often developе certain muscles of the face to such a degree as to impart to the countenance an expression of ill nature, when they are not in anger.

system have ceased to enlarge. "This phenomenon," says M. Itard, "is not very rare, even in the adult, especially among men given to study, or profound meditation, or who devote themselves, without relaxation, to the agitations of an unquiet and enterprising spirit. The head of *Bonaparte*, for instance, was small in youth, but acquired in after life, a development nearly enormous."*

"I would have the parent, therefore, understand that his child may be made to excel in almost any thing; that by increasing the power of certain organs through exercise, he can be made a prodigy for early mental or muscular activity. But I would have him at the same time, understand the conditions upon which this can be effected, and its consequences. I would have him fully aware, that in each case, unusual activity and power is produced, by extraordinary development of an organ; and especially that in early life, no one organ of the body can be long exercised, without the risk of most disastrous consequences. Either the over-excited and over-tasked organ itself will be injured for life, or the development of other and essential parts of the system will be arrested forever.

"From what has been said hitherto, we gather the following facts, which should be made the basis of all instruction; facts which I wish often to repeat. *The brain is the material organ by which all the mental faculties are manifested; it is exceedingly delicate, and but partially developed in childhood; over-excitement of it when in this state, is extremely hazardous.*"

In section third our author sets forth his views on the consequences which have resulted from inattention to the connexion between the mind and body, and attempts to show, that the best minds are not produced by early mental culture. We shall present our readers with the opening paragraphs.

"Teachers of youth, in general, appear to think, that in exciting the mind, they are exercising something totally independent of the body, some mysterious entity, whose operations do not require any corporeal assistance. They endeavor to accelerate to the utmost the movements of an extremely delicate machine, while most unfortunately they are totally ignorant of its construction. They only know that its action and power may both be increased for a while, by the application of a certain force; and when the action becomes deranged, and the power destroyed, they know not what is the difficulty, nor how it can be remedied. Fortunately they do not attempt to remedy it themselves, but call in the physician, who, if he affords any relief at all, does it by operating on a material organ. If medical men entertained the same views with teachers, they would, in attempting to restore a deranged mind, entrely overlook the agency of the body, and

* Dictionnaire des Sciences Medicales, Vol. 22. Art. Hydrocephale.

instead of using means calculated to effect a change of action in the brain, would rely solely upon arguments and appeals to the understanding. For if the mind may be cultivated independent of the body, why may it not also be remedied when disordered, without reference to the body?"

After reprobating the various means to which parents and teachers sometimes resort, for stimulating children to inordinate exertion of their mental faculties, our author observes:—

"I beseech parents, therefore, to pause before they attempt to make prodigies of their own children. Though they may not destroy them, by the measures they adopt to effect this purpose, yet they will surely enfeeble their bodies, and greatly dispose them to nervous affections. Early mental excitement will serve only to bring forth beautiful but premature flowers, which are destined soon to wither away, without producing fruit."

The whole of the chapter is deeply interesting. We shall venture on one extract more.

"The early history of the most distinguished men will I believe lead us to the conclusion, that early mental culture is not necessary, in order to produce the highest powers of mind. There is scarcely an instance of a great man, one who has *accomplished* great results, and has obtained the gratitude of mankind; who in early life received an education in reference to the wonderful labors which he afterwards performed. The greatest philosophers, warriors, and poets, those men who have stamped their own characters upon the age in which they lived; or who, as Cousin says, have been the "true representatives of the spirit and ideas of their time," have received no better education when young than their associates who were never known beyond their own neighborhood. In general, their education was but small in early life. *Self Education*, in after life, made them great, so far as education had any effect."

Section fourth is devoted to the opinions of distinguished physicians in different countries, on the influence of early mental cultivation on the minds and bodies of children. The mass of authority is voluminous, and merits the profoundest attention. We have only room for a specimen, and shall select a part of that, which is drawn from France.

"In France, the education of youth has engaged the attention of many of the most learned and distinguished men. Numerous treatises upon the subject have been published, urging the importance of physical education. M. Friedlander, in a late work dedicated to M. Guizot, recently one of the ministry, thus speaks of early instruction. "From the highest antiquity we have this rule, that mental instruction ought not to commence before the seventh year." M. Friedlander thinks this rule is correct, and says that our climate, which necessarily confines children much of the time within doors, has led to the idea of teaching them early, and thus making them prodigies. He gives the following table for the hours of rest and labor, which he says is adopted by many instructors.

| Age. | Hours of sleep. | Hours of exercise. | Hours of occupation. | Hours of repose. |
|---|---|---|---|---|
| 7 | 9 to 10 | 10 | 1 | 4 |
| 8 | 9 | 9 | 2 | 4 |
| 9 | 9 | 8 | 3 | 4 |
| 10 | 8 to 9 | 8 | 4 | 4 |
| 11 | 8 | 7 | 5 | 4 |
| 12 | 8 | 6 | 6 | 4 |
| 13 | 8 | 5 | 7 | 4 |
| 14 | 7 | 5 | 8 | 4 |
| 15 | 7 | 4 | 9 | 4 |

M. Ratier, in an essay on Physical Education of Children, which was crowned by the Royal Society of Bordeaux in 1821, thus speaks of early mental instruction. "The labor of the mind, to which some parents subject their children not only too soon, but in a wrong direction, is often the cause of their bad health, and causes nearly all those who are distinguished by precocity of the intellectual faculties, to perish prematurely; so that we seldom see a *perfect man;* that is one who exhibits an equilibrium of the Physical, Mental and Moral faculties." M. Julien, late editor of the Revue Encyclopedique, in his large and valuable work on *Physical*, *Moral*, and *Intellectual Education*, remarks,—"All the pages of this work repel the double reproach, of wishing to hasten the progress of the intellect, and obtain premature success, or retard the physical development of children, by neglecting the means necessary to preserve their health. We have constantly followed the principle of Tissot, who wished that infancy might be consecrated to those exercises which fortify the body, rather than to mental application which enfeebles and destroys it." Again he observes, "The course to be adopted with children for the first ten years of life, is neither to press or torment them; but by plays, exercise of the body, entire liberty wisely regulated, and good nourishment to effect the salutary and progressive development of the Physical, Moral and Intellectual faculties, and by continual amusement and freedom from chagrin, (which injures the temper of children) they will arrive at the tenth year without suspecting that they have been made to learn any thing: they have not distinguished between study and recreation; they have learned all they know freely, voluntarily and always in play. The advantages obtained by this course, are good health, grace, agility, gaiety and happiness; a character frank and generous, a memory properly exercised; a sound judgment, and a cultivated mind."

The preceding chapter, is devoted to the intensely interesting subject of insanity and nervous affections, as the offspring, in many cases, of mental cultivation and mental excitement.

Our author, from data drawn from his own state, Connecticut, supposes there cannot be less than 50,000 deranged persons in the United States, but this estimate we are disposed to consider much too high. Connecticut it seems has about 1,000, or one insane person for every 262 inhabitants! We feel convinced that in many other parts of the Union—in the West for example—the proportion of the insane to the sane must be much less; and, indeed, if there be a reality, as we think there is, in the opinion of our author, that excessive mental excitement, tends to produce insanity, we might expect, *a priori*, that most of the states would be less afflicted with this malady, because they make less effort at early education than Connecticut.

"In view of these few brief facts respecting *Insanity*, we are forced to believe, that among the causes of the great prevalence of this disease in this country, are the following:—

"First, Too constant and too powerful excitement of the mind, which the strife for wealth, office, political distinction, and party success, produces in this free country.

"Second, The predominance given to the nervous system, by too early cultivating the mind and exciting the feelings of children.

"Third, Neglect of Physical education, or the equal and proper development of all the organs of the body.

"Fourth, The general and powerful excitement of the female mind. Little attention is given in the education of females, to the physiological differences of the sexes. Teachers seldom reflect, that in them the nervous system naturally predominates; that they are endowed with quicker sensibilities than men, and have imaginations far more active, that their emotions are more intense, and their senses alive to more delicate impressions; and therefore require great attention; lest this exquisite sensibility, which, when properly and naturally developed, constitutes the greatest excellence of women, should either become *excessive* by too strong excitement, or suppressed by misdirected education. If here was the proper place, it would be easy to show that efforts to make females excel in certain qualities of mind which in men are considered most desirable,—to make them as capable as men, of long continued attention to abstract truths, would be to act contrary to the dictates of nature as manifested by the organization, and would tend to suppress all those finer sensibilities, which render them in every thing that relates to sentiment and affection far superior to men."

The sixth chapter is devoted to moral education, and in the seventh our author attempts to show, that timely and proper cultivation of the mind is not injurious, but beneficial to health and favorable to longevity; in the last, he treats of the influence of mental cultivation in producing dyspepsia, which he believes, in most cases to *arise* from an irritation of the brain. We cannot follow him through his ingenious reasonings on this topic, but hope our readers will examine the subject for themselves. The whole book is every way worthy of being introduced into the library of every physician, and we should like to see an edition of it published in the West, at a time when the subject of education is attracting more attention than has heretofore been bestowed upon it. Dr. Brigham is evidently a man of sound sense and industrious research.

We have inadvertently extended our notice of Dr. Brigham's book, till we have but little room for an account of Mr. Weld's Report. The object of this report, is to demonstrate by facts and arguments, that *Manual Labor* should be enjoined as a mode of exercise in all our academies and colleges. Mr. W. was himself educated in the Manual Labor Seminary of Oneida, New-York; and, as agent of the Society, travelled for a year, through the northern and western States, making observations, and collecting, on this subject, the experience and opinions of many eminent teachers and physicians, which he has skilfully embodied in his very able report to the Society. In this Report he has with great ability considered the necessity of physical exercise to the health and intellectual development of students and literary men; and shown, we think triumphantly, that nothing can be relied upon but manual labor, required as a part of their discipline. Left to themselves, students will not, in general, take exercise; it must be enjoined upon them for they know not its value, and no other than manual labor can be enforced. As far as practicable it should be in the field, after that, in the workshop, where it should be of such kinds as keep them on their feet, exercise all their muscles, and produce fatigue. Mr. Weld has made a high and just estimate of the beneficial effects of labor, thus prosecuted, to the consitutions and health of all who persevere in it; and has, we think, shown most conclusively, that it not merely comports with, but actually promotes the finest cultivation of the faculties and affections of the mind. As physicians we are not immediately interested in the system, beyond this point; but Mr. W. as a political economist has gone further, and shown that students may be made to contribute largely to their support during the period of study; and, consequently, that if Manual Labor Seminaries were generally instituted, a much greater number would receive a competent and useful, if not a liberal education, than now do. Under this view, they become a national object of the highest interest, as they would contribute, most efficiently, to elevate into society a great number of valuable young men, who, under the present system, are doomed to remain in ignorance and obscurity. Thus they would greatly swell the amount of national intellect. But in this result the medical profession, in reference to its own dignity and usefulness, has a deep and direct interest. It is well known to all who are practically conversant with medical instruction, that by far the greater number of those who are put to the study of medicine, are young men from the middle walks of life, who have not had the means of acquiring an academical education and are entirely deficient in preparatory learning. Most of these, if manual labor academies had been within their reach, would have made themselves good scholars. Had such schools been instituted twenty years ago, the medical profession, in the United States, would have been very different from what it now is; and whenever they shall be established, they will be found to exert an early and most beneficial influence on the character of our physicians.

But we must lay before our readers one or two specimens of Mr. Weld's very able report. The first that we shall select relates to the reciprocal influence of the mind and body:—

"The effects produced by different states of the mind upon the body are equally sudden and powerful. Plato used to say, that "all the diseases of the body proceed from the soul." The expression of the countenance *is mind visible. Bad news* weakens the action of the heart, oppresses the lungs, destroys appetite, stops digestion, and partially suspends all the functions of the system. An emotion of shame flushes the face; fear blanches it; joy illuminates it, and an instant thrill electrifies a million nerves. Surprise spurs the pulse into a gallop. Delirium infuses giant energy. Volition commands, and hundreds of muscles spring to execute. Powerful emotion often kills the body at a stroke. Chilo, Diagoras, and Sophocles, died of joy at the Elean games. The news of a defeat killed Philip V. One of the popes died of an emotion of the ludicrous, on seeing his pet monkey robed in pontificals, and occupying the chair of state. Muley Moluck was carried upon the field of battle in the last stages of an incurable disease. Upon seeing his army give way, he leaped from the litter, rallied his panic stricken troops, rolled back the tide of battle, shouted victory, and died. The door-keeper of Congress expired upon hearing of the surrender of Cornwallis. Eminent public speakers have often died, either in the midst of an impassioned burst of eloquence, or when the deep emotion that produced it had suddenly subsided. The late Mr. Pinckney of Baltimore, Mr. Emmet, of New York, and the Hon. Ezekiel Webster, of New-Hampshire, are recent instances. Lagrave, the young Parisian, died a few months since, when he heard that the musical prize for which he had competed was adjudged to another. The recent case of Hills in New-York is fresh in the memory of all. He was apprehended for theft, taken before the police, and though in perfect health, mental agony forced the blood from his nostrils. He was carried out, and died.

"The experience of every day demonstrates that the body and mind are endowed with such mental susceptibilities, that each is alive to the slightest influence of the other. What is the common sense inference from this fact? Manifestly this: that the body and the mind *should be educated together*. The states of the body are infinitely various. All these different states differently affect the mind. They are causes, and their effects have all the variety which mark the causes that produce them. If then different conditions of the body differently affect the mind, some electrifying, and others paralyzing its energies, what duty can be plainer than *to preserve the body in that condition which will most favorably affect the mind.* If the Maker of both was infinitely wise, then the highest *permanent* perfection of the mind can be found only in connection with the most healthful state of the body. Has infinite wisdom established laws by which the best condition of the mind is *permanently* connected with any other than the best condition of the body? When all the bodily functions are perfectly performed, the mind must be in a better state than when these functions are imperfectly performed. And now I ask, is not that system of education fundamentally defective, which makes no provision for putting the body in its best condition, and for keeping it in that condition?—a system which expends its energies upon the mind alone, and surrenders the body either to the irregular promptings of perverted instinct, or to the hap-hazard impulses of chance or necessity?—a system which aims solely at the development of mind, and yet overlooks those very principles which are indispensable to produce that development, and transgress those very laws which constitute the only groundwork of rational education?

Such a system sunders what God has joined together, and impeaches the wisdom which pronounced that union good. It destroys the symmetry of human proportion, and makes man a monster. It reverses the order of the constitution; commits outrage upon its principles; breaks up its reciprocities; makes war alike upon physical health and intellectual energy, dividing man against himself; arming body and mind in mutual hostility, and prolonging the conflict until each falls a prey to the other, and both surrender to ruin.

Almost at random we shall transcribe another specimen, in which our author pleasantly and pungently hits off the folly of the governors of our literary institutions, who recognize and recommend to the pupils necessity of exercise, without providing the means:

"Shall we plod through the same process forever? Shall we trudge on in this profitless round of exhortation and remonstrance, doling out the same old lamentation, and reading at every step the same stale homily upon exercise, which rarely if ever made a practical convert. Shall we play the fool any longer, by repeating an experiment in the face of ten thousand failures under the same circumstances, and when in the very laws of human nature there is 'confirmation strong' that every *future* effort will result in failure? Let us suppose a case.

Suppose all our existing institutions were established solely for the education of the *body*. Suppose each had a complement of teachers, full courses of lectures and experiments, merely for promoting the beauty and strength, symmetry and perfect action of the *body*—all the requisitions leaving utterly out of sight the whole circle of science and literature. Suppose three hours of relaxation from these exercises are allowed the students daily. Suppose moreover, they are told they have *minds*, and are eloquently urged to devote these three hours each day to mental improvement. But not a single facility is furnished for the accomplishment of this object. No apparatus, lectures or experiments, no libraries, no cabinets, no recitations, no teachers, no requisitions for study, and no regulations designed to afford the least *aid or countenance* to the student in the culture of his mind; and not only so, but these three hours of leisure are divided into four or five portions, and are distributed over the day, and so *entangled with other arrangements* as to make it exceedingly difficult to employ them in mental cultivation. Thus, instead of receiving any encouragement to educate his mind, all the exercises of the institution are arranged in such a manner that the student is harassed, subjected to great inconvenience in all his efforts to acquire knowledge, finds himself unsustained by the example of his teachers, who are most zealously engaged in the improvement of their *bodies* alone, public sentiment is against him, and he becomes disheartened, and rather than carry on a contest without auxiliary or ally, he surrenders at discretion, and lounges away his leisure, in conformity with the fashion around him. Still, once a quarter, perhaps, he is dos'd with a lecture upon the *importance of educating his mind*, and exhorted to provide himself with all the requisite facilities, and set about the work in earnest during his leisure hours!! What would such appeals avail? They would excite unmingled contempt and disgust; and the more eloquently they were urged, and the oftener repeated, the more they would be regarded as downright mockery and insult. Well might the student exclaim, 'What! will you taunt the misery of a starving man, by exhorting him to eat while you deny him bread, or will you put him in fetters and handcuffs and then place food before him, merely to mock his anguish by tempting his appetite? Do you exhort us to store our minds with science, and yet deny us the means of its acquisition? Give us access to libraries and cabinets, laboratories and apparatus, experiments and lectures; furnish us with teachers, make *requisitions* for study and recitation, for composition, discussion and declamation; make specific regulations, have a *system*—then we will believe you *honest* and *in earnest*.'

> "*Conduct* hath the loudest tongue. The voice
> Is but an instrument on which a man
> May play what tune he pleases. In the *deed*,
> The unequivocal, authentic *deed*,
> We find *sound argument*."

The application of this supposition to the case before us is manifest. Look at our institutions as they are. All not only admit the importance of exercise, but advise and urge students to take it regularly and in considerable quantities, and yet have not made *a tittle of provision for it;* have furnished not a single facility; have made no arrangement of studies and recitations, calculated in the least to favor it. All their regulations are profoundly silent about health and physical improvement; passing over the body as if beneath their notice. leaving the whole matter of physical education to the supervision of chance and accident, to be regulated by freaks and fantasies, under the dictation of ignorance and caprice. Thus our institutions practically nullify their own precepts, by withholding every facility for reducing them to practice. They render their exhortations void, and their remonstrances of none effect, by refusing to furnish those means, and to adopt that system, without which *they must of necessity become a dead letter.*"

We must bring this *lengthy* article to a close, by expressing the hope, that both the books under our notice will be purchased and read extensively. Dr. B. has pointed out the disastrous effects of early excessive application to study—Mr. W. has shown us how they may be averted. Few students of any age or of either sex, would suffer from hard study, if they could be induced to follow his excellent precepts. Indeed, we look upon Mr. Weld's book as one of the most valuable in its tendencies, which the age has produced. Should it promote as it ought, the union of manual labor, with academical and university study, it will constitute an epoch in society—multiplying to a great extent the number of educated men—disenthralling genius from the shackles of poverty—averting the infirmities of men of letters—dispersing the gloom which so often creeps over their minds, and favoring that fulness of years and knowledge, which is the natural effect of combined intellectual and physical cultivation.

